# Integrated Transcriptomics and Metabolomics Analysis of the Formation Mechanism of Low Bitterness and Astringency in “Huangjinya” Tea

**DOI:** 10.3390/plants15162508

**Published:** 2026-08-19

**Authors:** Linghui Wang, Quan Xu, Miao Xu, Xin Tong, Lianxin Liu, Ding Tang, Suyu Xia, Ji Huang, Wei Li, Xia Zhao, Qiulan Huang

**Affiliations:** 1 Faculty of Agriculture, Forestry and Food Engineering, Yibin University, Yibin 644001, China; zjwwlh08@126.com (L.W.); zhoujinwei08@126.com (Q.X.); 15528988641@163.com (M.X.); m18283035417@163.com (X.T.); m18380825300@126.com (L.L.); 17313911658@163.com (D.T.); 15775290325@163.com (S.X.); huangjirh@163.com (J.H.); lw816816@163.com (W.L.); 2 Tea Resources Utilization and Quality Testing Key Laboratory of Sichuan Province, Chengdu 611130, China

**Keywords:** *Camellia sinensis*, low astringency flavor, metabolome, transcriptomics, catechin accumulation, gene expression regulation

## Abstract

The flavor variation among different tea plant cultivars significantly influences their economic value. ‘Huangjinya’ (HJY), a typical light-sensitive etiolated tea cultivar, is prized for its fresh and mellow taste with low bitterness and astringency, and commands a high market value, serving as excellent germplasm for breeding superior-flavor tea varieties. Previous studies have largely focused on the mechanisms underlying fresh and umami tastes, whereas the regulatory mechanisms underlying bitterness and astringency remain poorly understood. In this study, two tea plant (*Camellia sinensis* (L.) O. Kuntze) cultivars with distinct bitterness and astringency phenotypes, namely ‘Fuxuan 9’ (FX) and HJY, were used to investigate the formation mechanism of reduced bitterness and astringency through integrated metabolomic and transcriptomic analyses. Metabolomic profiling identified 601 differentially accumulated metabolites between the two cultivars, which were significantly enriched in the flavonoid biosynthesis pathway. Notably, although the total flavonoid content was higher in HJY, the accumulation of non-esterified catechins (C, EC, EGC), which are key contributors to bitterness, was significantly lower than in FX. By contrast, the levels of esterified catechins (EGCG, GCG) showed no marked differences between cultivars. This distinct accumulation pattern provides a metabolic basis potentially associated with the reduced bitterness and astringency in HJY. Transcriptomic analysis revealed that the catechin biosynthetic gene *CsLAR* was significantly down-regulated in HJY, consistent with the reduced accumulation of non-esterified catechins. Promoter cloning and functional validation confirmed that the *CsLAR* promoters in both cultivars were active, which differed by only one regulatory element. Under shading treatment, *CsLAR* expression in HJY was more sensitive to light and decreased more markedly, suggesting that the expression of *CsLAR* may be regulated by transcription factors. Furthermore, 385 differentially expressed transcription factors were identified, with predicted binding sites in the *CsLAR* promoter region, implying their potential involvement in modulating catechin accumulation through modulation of *CsLAR* expression. Together, these multi-omics findings reveal molecular features potentially contributing to the low bitterness and astringency of HJY, providing a theoretical basis and candidate regulators for breeding tea cultivars with improved flavor profiles.

## 1. Introduction

The tea plant is an economically important crop that is widely cultivated worldwide. As one of the three most popular non-alcoholic beverages, tea contains abundant health-promoting compounds, such as flavonoids, theanine, and caffeine, which contribute to its flavor profile and provide multiple health benefits [1]. Furthermore, tea quality and flavor vary depending on the specific tea variety [2]. Given its considerable health and economic importance, increasing research attention has been directed toward understanding and improving tea quality.

Flavonoids are key metabolites affecting tea quality, accounting for 20–40% of the dry weight [3]. The metabolites include catechins (flavan-3-ols), flavonols, flavonol glycosides, chalcones, among others [4], and they exhibit distinct accumulation patterns observed across different varieties. Catechins, comprising over 60% of total flavonoids, are principal contributors to tea bitterness and exhibit multiple bioactivities, including anti-cancer, antioxidant, and cardiovascular protective effects [5,6]. Based on structural features, catechins are categorized into ester-type and non-ester-type forms. Non-esterified catechins include catechin (C), epicatechin (EC), and epigallocatechin (EGC), and primarily contribute to bitter sensation with mild astringency, whereas esterified catechins, such as epigallocatechin gallate (EGCG) and epicatechin gallate (ECG), confer intensive persistent astringency and also participate in bitterness formation [7,8]. Nevertheless, excessive accumulation of ester-type catechins can significantly reduce tea quality [2,9]. The biosynthetic pathways of catechins have been largely elucidated and involve crosstalk among phenylpropanoid, flavonoid, and shikimate pathways. Specifically, non-esterified catechin biosynthesis is catalyzed by a series of key enzymes, including chalcone synthase (CHS), chalcone isomerase (CHI), flavonoid 3-hydroxylase (F3H), flavonoid 3′,5′-hydroxylase (F3′5′H), dihydroflavonol 4-reductase (DFR), leucoanthocyanidin reductase (LAR), anthocyanidin synthase (ANS), and anthocyanidin reductase (ANR) [10,11]. The synthesis of ester-type catechins is primarily mediated by galloyl-1-O-β-D-glucosyltransferase (UGGT) and 1-O-galloyl-β-D-glucose O-galloyltransferase (ECGT) [12]. Variations in the accumulation patterns of ester and non-ester catechins significantly influence tea flavor. Nevertheless, the molecular mechanisms underlying catechin component distribution across different tea varieties remain poorly understood.

Albino teas, a common phenotypic variation in tea plants, are characterized by white or yellow shoot phenotypes under specific environmental conditions [13]. Compared with conventional green-leaf tea, albino teas generally exhibit lower bitterness and astringency flavors. HJY, a representative albino mutant with yellow leaves, possesses high market value due to its enhanced sweetness and reduced bitterness. Previous studies on HJY have focused on leaf coloration mechanisms and developmental processes. Its seasonal regreening has been attributed to the inhibition of chlorophyll biosynthesis mediated by *CsHY5* gene expression [14], and leaf color is also influenced by environmental factors such as light quality and shading [15,16,17]. Recent advances in omics technologies have shifted research attention to secondary metabolites. Several studies have highlighted the contribution of theanine accumulation to the umami taste of yellow leaf tea [18,19,20]. However, the low-bitterness trait of HJY remains of particular interest. Studies have shown that tea bitterness is closely associated with catechin accumulation [2]. Nevertheless, limited research has been conducted on the synthesis and metabolism of catechins in HJY, and the underlying molecular mechanisms remain poorly understood.

Although studies have investigated tea metabolism and flavor formation, the key taste components and regulatory mechanisms underlying the characteristic “high freshness and low bitterness” of albino tea remain unclear. In this study, we conducted integrated metabolomic and transcriptomic analyses to compare the albino tea cultivar HJY with the conventional green-leaf cultivar FX, aiming to elucidate differences in metabolite accumulation and their molecular regulation, and to identify metabolic features that may be associated with the reduced bitterness and astringency of HJY. Our research elucidates the metabolic basis and regulatory mechanisms underlying its low-bitterness trait, and identifies the key functional genes involved. These findings enhance our understanding of the fundamental differences in quality-related compound accumulation between HJY and conventional green-leaf tea. Furthermore, they provide a theoretical basis and new insights for screening, developing, and breeding tea plant germplasm with potentially improved flavor profiles.

## 2. Materials and Methods

### 2.1. Plant Materials

Two tea cultivars, FX and HJY, were selected for this study. Both were cultivated in the same tea plantation under consistent growth conditions. Buds at comparable developmental stages were collected, immediately frozen in liquid nitrogen, and stored at −80 °C. Three independent biological replicates were randomly sampled from more than 50 tea plants.

### 2.2. Detection of Total Amino Acids and Flavonoids

The total amino acid content was determined according to the method described by Wu et al. [21]. Briefly, approximately 5 g of fresh tea leaves were extracted with 90 mL of distilled water in a boiling water bath for 45 min. After cooling, the extract was diluted to a final volume of 100 mL with distilled water. The total amino acid content was then calculated using glutamic acid as the standard.

The total flavonoid content was determined by the colorimetric method based on the protocol of Zhao et al. [22]. Quantitative analysis was performed using a calibration curve established with rutin standard. The procedure was as follows: an aliquot of the extract was mixed with 5% NaNO_2_ solution and allowed to stand for 6 min. Then, 10% Al(NO_3_)_3_ solution was added, and the mixture was allowed to stand for another 6 min. Finally, 1 mol/L NaOH was added, and the absorbance was immediately measured at 510 nm using a spectrophotometer (Unico (Shanghai) Instruments Co., Ltd., Shanghai, China). To ensure data reliability, each extract sample was determined in triplicate.

### 2.3. Transcriptome Sequencing and Analysis

Bud samples from both tea cultivars stored at −80 °C were ground in liquid nitrogen. Total RNA was extracted using TRIzol reagent following the manufacturer’s instructions. cDNA libraries were constructed with high-quality RNA and sequenced on the Illumina platform by MetWare Biotechnology Co., Ltd. (Wuhan, China) (http://www.metware.cn/). Raw sequencing reads were filtered to obtain clean data, which were then aligned to the reference genome of the tea plant. The mapped reads were used for the analysis of alternative splicing, novel transcript identification, and gene structure refinement. Functional annotation and enrichment analyses of differentially expressed genes (DEGs) were performed using Gene Ontology (GO) and Kyoto Encyclopedia of Genes and Genomes (KEGG) databases [23].

### 2.4. Metabolite Detection and Analysis

Widely targeted metabolomics analysis was conducted by MetWare Biotechnology using a UPLC-MS/MS system. Briefly, 50 mg of freeze-dried powder was extracted with 1.2 mL of 70% aqueous methanol at room temperature. The extract was vortexed for 30 s every 30 min (six times in total) to ensure thorough mixing. After centrifugation, the supernatant was filtered through a 0.22-μm microporous membrane prior to analysis. Chromatographic separation was performed on an Agilent SB-C18 column (1.8 μm, 2.1 mm × 100 mm) using a Shimadzu Nexera X2 UPLC system (Shimadzu Corporation, Kyoto, Japan) at a flow rate of 0.35 mL/min and a column temperature of 40 °C. The mobile phase consisted of 0.1% formic acid in water (A) and 0.1% formic acid in acetonitrile (B), with the following gradient elution: 5–95% B over 0–9 min, held at 95% B for 1 min, decreased to 5% B from 10.0 to 11.1 min, and re-equilibrated at 5% B until 14 min. The injection volume was 4 μL.

Mass spectrometric analysis comprised two independent analytical workflows. Metabolite identification was performed using an AB Sciex 6600 Q-TOF mass spectrometer (AB Sciex LLC, Marlborough, MA, USA) equipped with an electrospray ionization (ESI) source. Data acquisition was carried out using information-dependent acquisition (IDA) mode. The optimized source parameters were as follows: ion spray voltage, +5500 V for positive-ion mode and −4500 V for negative-ion mode; source temperature, 500 °C; nebulizer gas (GS1), 50 psi; heater gas (GS2), 50 psi; curtain gas, 25 psi; and declustering potential (DP), ±60 V for positive and negative modes, respectively. The TOF-MS survey scan was performed over the mass range of 100–1250 Da with an accumulation time of 200 ms. IDA-triggered product ion scans were acquired over the mass range of 50–1250 Da with an accumulation time of 50 ms. The collision energy (CE) was set at ±30 eV with a collision energy spread (CES) of 15 eV. Dynamic background exclusion was enabled, with an intensity threshold of 100 counts per second (cps), isotope exclusion within ±4 Da, and mass tolerance of 50 parts per million (ppm). The mass resolution of the 6600 Q-TOF was ≥35,000 (FWHM) at m/z 956.

For targeted relative quantification of metabolites, an AB Sciex 4500 QTRAP triple quadrupole mass spectrometer (AB Sciex LLC, Marlborough, MA, USA) was employed as described previously [24,25]. The optimized source parameters were as follows: ion spray voltages, +5500 V and −4500 V for positive and negative modes, respectively; source temperature, 550 °C; nebulizer gas (GS1) and heater gas (GS2), 50 and 60 psi, respectively; curtain gas, 25 psi; and collision-activated dissociation (CAD) set to high. Data acquisition and peak integration were performed using Analyst 1.6.3 and MultiQuant 3.0.3 software, respectively. Metabolite identification was conducted against the MetWare Database (MWDB), with mass tolerances of 20 ppm for both MS1 and MS2 and a retention time tolerance of 0.2 min. Raw peak areas were adopted for relative quantification, with missing values filled with 9.

### 2.5. Plasmid Construction and Tobacco Transient Transformation

The plant expression vector pCAMBIA3301 was used for transient expression assays in tobacco leaves. The *CsLAR* promoter sequence was amplified using primers P-*CsLAR*-F/R and KOD One™ PCR Master Mix (Toyobo Co., Ltd., Osaka, Japan). The resulting PCR product was digested and directionally cloned into the PstI/NcoI restriction sites to drive *GUS* expression. The constructed pCAMBIA3301-P-*CsLAR*::*GUS* plasmid was verified by enzymatic digestion and sequencing. The confirmed construct was then introduced into tobacco leaves via *Agrobacterium tumefaciens* strain GV3101 using the vacuum infiltration method. Promoter activity was assessed by histochemical GUS staining.

### 2.6. Quantitative Real-Time PCR (qRT-PCR) Analysis

Plant samples were ground with liquid nitrogen, and total RNA was extracted using a Plant Total RNA Isolation Kit (Tiangen Biotech Co., Ltd., Beijing, China). A PrimeScript RT kit (Toyobo Co., Ltd., Osaka, Japan) was used to synthesize the first-strand cDNA. Gene-specific primers were designed using Primer Premier 5 software, and the primer sequences are listed in Table 1. Quantitative real-time PCR was performed using TransStart^®^ Green qPCR SuperMix (TransGen Biotech Co., Ltd., Beijing, China) on a CFX96 real-time PCR system (Bio-Rad Laboratories, Inc., Hercules, CA, USA), with *GAPDH* (CSS0008920) used as the internal reference gene. Three replicates were performed for each plant group.

### 2.7. Statistical Analysis

Data were expressed as the mean ± standard deviation (SD) from three independent biological replicates. Statistical analyses were performed with SPSS 19.0, and differences were considered statistically significant at *p* < 0.05. GraphPad Prism 5 and TBtools-II (v2.486) were used to generate the figures.

## 3. Results

### 3.1. Analysis of Total Amino Acid and Flavonoid Contents in FX and HJY

**Figure 1:**
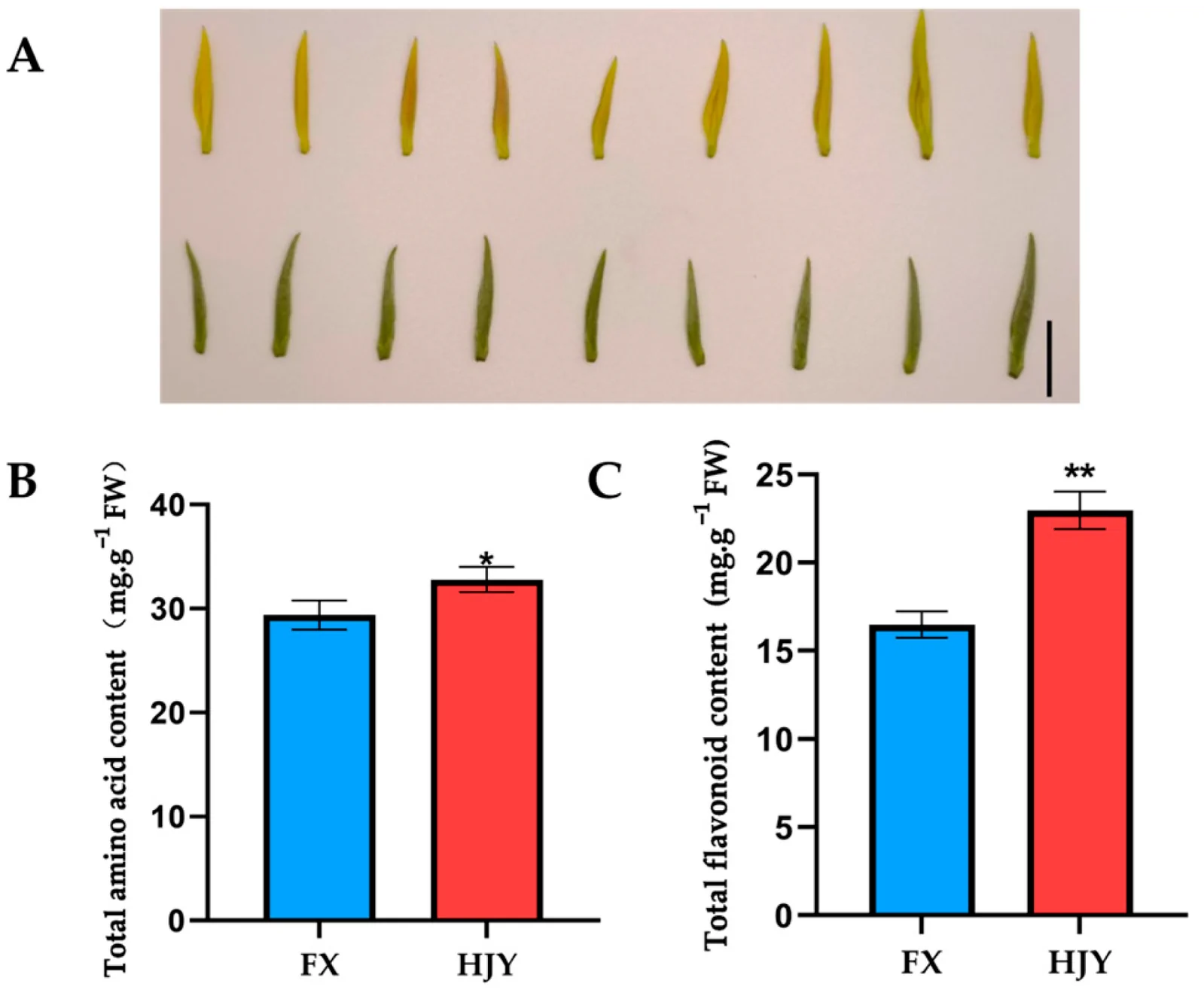
Phenotypic and biochemical comparisons between HJY and FX. (**A**) Phenotypes of representative buds from FX and HJY. Scale bar = 1 cm; (**B**) Total amino acid content in FX and HJY; (**C**) Total flavonoid content in FX and HJY. Data represent means ± standard deviation of three biological replicates. Asterisks indicate significant differences between the two cultivars by one-way ANOVA followed by Dunnett’s test (* *p* < 0.05, ** *p* < 0.01).

Amino acids and flavonoids are key substances influencing tea flavor. The results showed that the total amino acid content in HJY was 32.79 mg/g, which was significantly higher than that in FX, representing an increase of 11.61% (Figure 1B). Meanwhile, the total flavonoid content of HJY was 22.96 mg/g, which was 28.17% higher than that in FX (Figure 1C).

### 3.2. Metabolomics Analysis Revealed the Differential Metabolites Between FX and HJY

**Figure 2:**
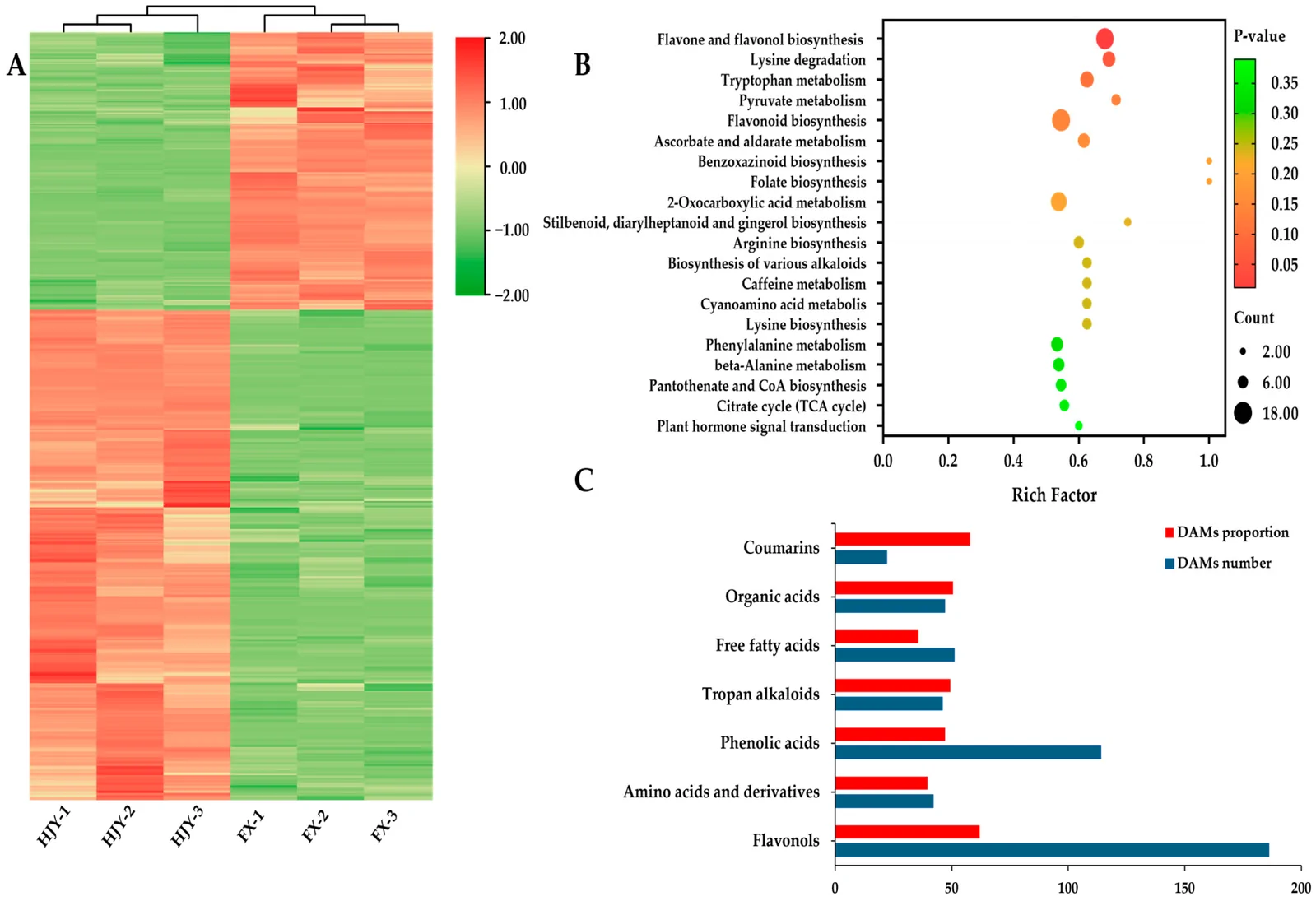
The differentially accumulated metabolites between FX and HJY. (**A**) Cluster heatmap of differential metabolites, with red and green indicating high and low relative abundance, respectively; (**B**) KEGG pathway enrichment analysis of DAMs between FX and HJY; (**C**) Number and proportion of DAMs in different metabolite classes.

To investigate the differential accumulation of metabolites between the two tea cultivars, a widely targeted metabolomics approach was performed using the UPLC-MS/MS platform. Cluster analysis of samples showed that replicates from the same cultivar clustered closely together, indicating high reproducibility among biological replicates (Figure 2A). A total of 1228 metabolites were identified, among which 601 were differentially accumulated metabolites (DAMs), including 383 upregulated and 218 downregulated metabolites, demonstrating significant differences in metabolite accumulation between the two cultivars (Table 2). KEGG pathway enrichment analysis revealed that the DAMs were primarily enriched in pathways related to flavonoid and flavonol biosynthesis, lysine degradation, and tryptophan metabolism (Figure 2B). Notably, the flavonoid and flavonol biosynthesis pathways contained the highest number of differentially accumulated metabolites.

The DAMs were categorized into seven major categories, including flavonoids, phenolic acids, amino acids and derivatives, tropane alkaloids, lipids, organic acids, and nucleotides and derivatives. The flavonoids and phenolic acids were the most abundant, comprising 186 and 114 metabolites, respectively (Figure 2C). Differentially accumulated flavonoid metabolites account for approximately 62% of the total flavonoid metabolites, representing the highest proportion among the seven major metabolites. Based on the enrichment patterns of KEGG pathways and the compositional analysis of DAMs, these results suggest that the taste differences between the two cultivars are closely associated with differentially accumulated flavonoids.

### 3.3. Differential Accumulation of Flavonoid Compounds Between FX and HJY

**Figure 3:**
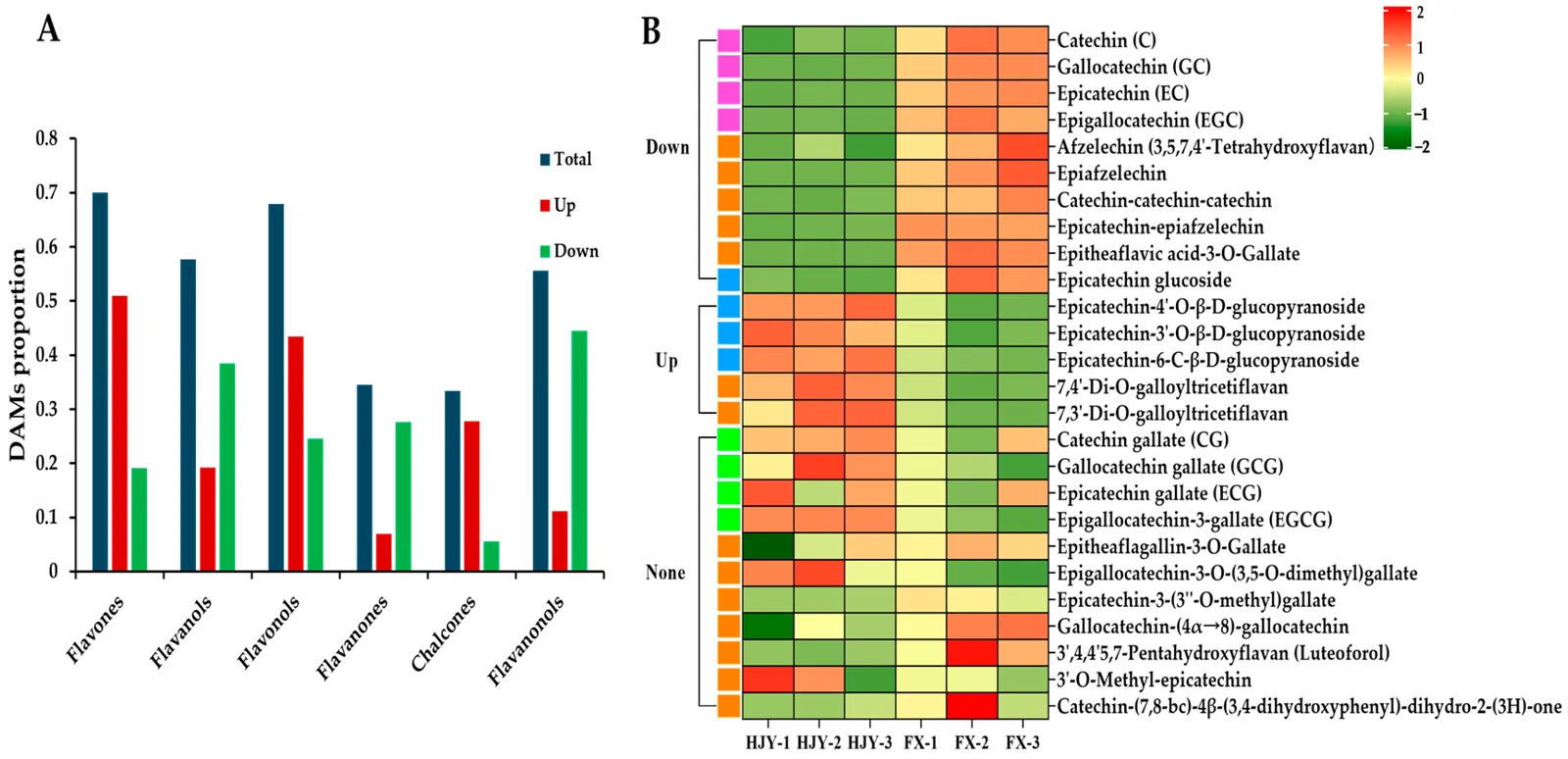
Differential accumulation of flavonoid compounds between FX and HJY. (**A**) Proportion of DAMs in various flavonoid subclasses; (**B**) Heatmap illustrating comparative accumulation of catechin compounds in FX and HJY. In heatmap, green denotes low relative metabolite abundance and red denotes high abundance. Left-side color blocks mark metabolite chemical categories: pink for non-esterified catechins, blue for glycosylated catechin derivatives, green for esterified catechins, orange for other flavanol derivatives.

To elucidate the mechanisms underlying the flavor differences between the two tea cultivars, we focused on the accumulation patterns of flavonoids. Metabolomic analyses identified 115 up-regulated and 71 down-regulated flavonoid compounds in HJY, suggesting that this cultivar possessed a stronger capacity for flavonoid accumulation. Flavonoids encompass multiple subclasses, among which flavanols (e.g., catechins) are the primary contributors to the bitter and astringent taste of tea infusions. Metabolomic comparison of the two tea varieties demonstrated a significant reduction in flavanol compound accumulation, with the proportion of down-regulated flavanol accumulation categories reaching 38.46% (Figure 3A). This reduction may represent a key factor potentially contributing to the attenuated bitterness and astringency of HJY tea infusions.

Catechins can be categorized into esterified and non-esterified types. Comparative analysis showed that all non-esterified catechins (C, GC, EC, and EGC) exhibited significantly lower levels in HJY than in FX. In contrast, no statistically significant differences were observed in the accumulation of esterified catechins (EGCG, GCG, CG, and ECG) between the two cultivars (Figure 3B).

### 3.4. KEGG Analysis of DEGs in FX and HJY

**Figure 4:**
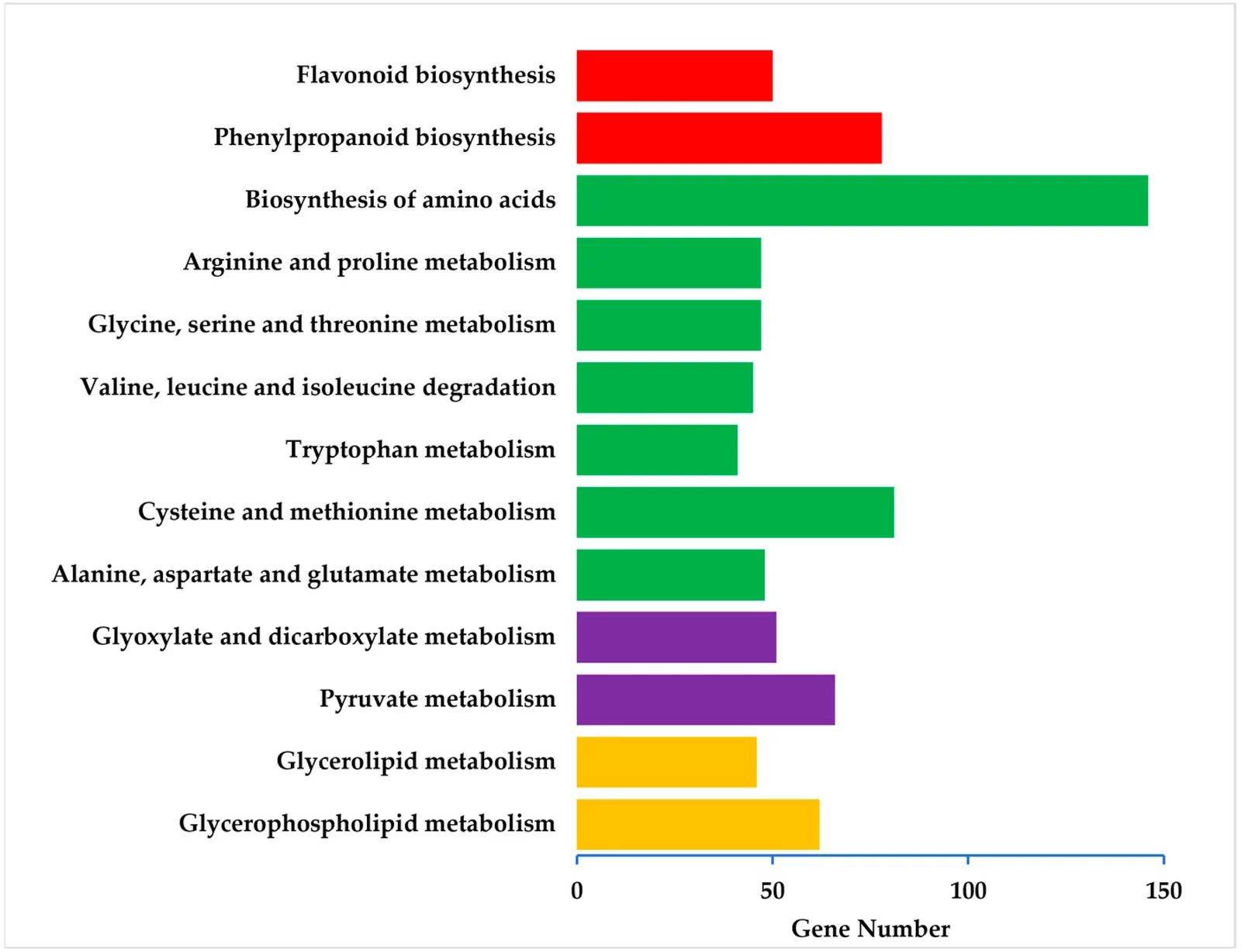
KEGG pathway enrichment analysis of DEGs between FX and HJY. Red bars represent flavonoid biosynthesis and metabolism, green bars represent amino acid metabolism, purple bars represent organic acid metabolism, and orange bars represent lipid metabolism.

To investigate the molecular mechanisms underlying the differential accumulation of metabolites between the two tea varieties, transcriptome sequencing was performed on RNA extracted from tender buds using the Illumina platform. A total of 9099 DEGs were identified between FX and HJY, of which 4368 were up-regulated and 4731 were down-regulated in HJY relative to FX (Table 3). KEGG enrichment analysis was performed on the DEGs. Among the top 50 enriched KEGG pathways ranked by the number of DEGs, significant enrichment was observed in multiple metabolic pathways, including flavonoid biosynthesis, phenylpropanoid biosynthesis, amino acid metabolism, organic acid metabolism, and lipid metabolism (Figure 4). These transcriptional-level findings were consistent with the results obtained from widely targeted metabolomic profiling, further corroborating the metabolic differences in various metabolite accumulations observed by metabolomics. Therefore, the DEGs involved in the flavonoid and phenylpropanoid biosynthetic pathways can serve as a basis for elucidating the molecular mechanisms underlying the differential accumulation of flavonoid metabolites between the two materials.

### 3.5. Differential Expression of Genes Involved in Flavonoid Biosynthesis Between FX and HJY

**Figure 5:**
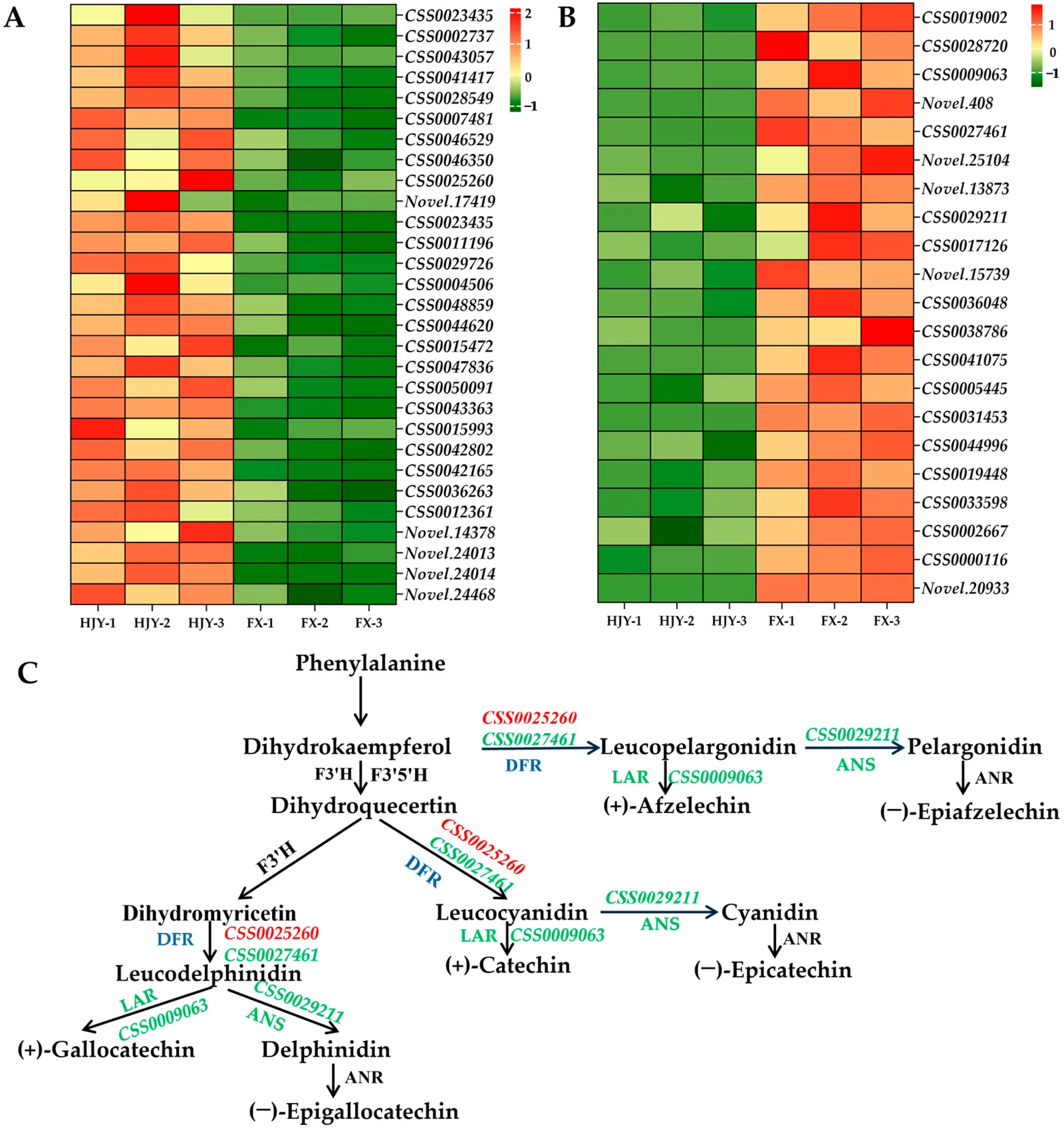
Analysis of DEGs involved in the flavonoid biosynthesis pathway. (**A**) Hierarchical clustering heatmap of up-regulated DEGs associated with flavonoid biosynthesis. Red and green indicate high and low expression levels, respectively. (**B**) Hierarchical-clustering heatmap of down-regulated DEGs associated with flavonoid biosynthesis. Red and green indicate high and low expression levels, respectively. (**C**) Regulation of DEGs in the catechin synthesis pathway.

Further analysis of the flavonoid biosynthesis pathway revealed 50 DEGs, of which 29 were up-regulated and 21 were down-regulated in HJY relative to FX (Figure 5A,B). Notably, *LAR* (gene ID: CSS0009063), a key gene directly involved in catechin synthesis, was significantly down-regulated in HJY. This expression pattern was consistent with the metabolomic data, which showed reduced catechin accumulation in this cultivar. In contrast, the expression level of *ANR*, a key gene associated with epicatechin synthesis, did not show a significant difference between the two varieties. Another catechin biosynthesis-related gene, *ANS*, exhibited significant down-regulation in HJY (Figure 5C). Based on the correlation between gene expression profiles and metabolite accumulation, the differential expression of *LAR* may represent a key regulatory factor contributing to the variation in catechin biosynthesis between the two cultivars.

### 3.6. Analysis of the CsLAR Promoter

#### 3.6.1. Activity Analysis of the *CsLAR* Promoter

**Figure 6:**
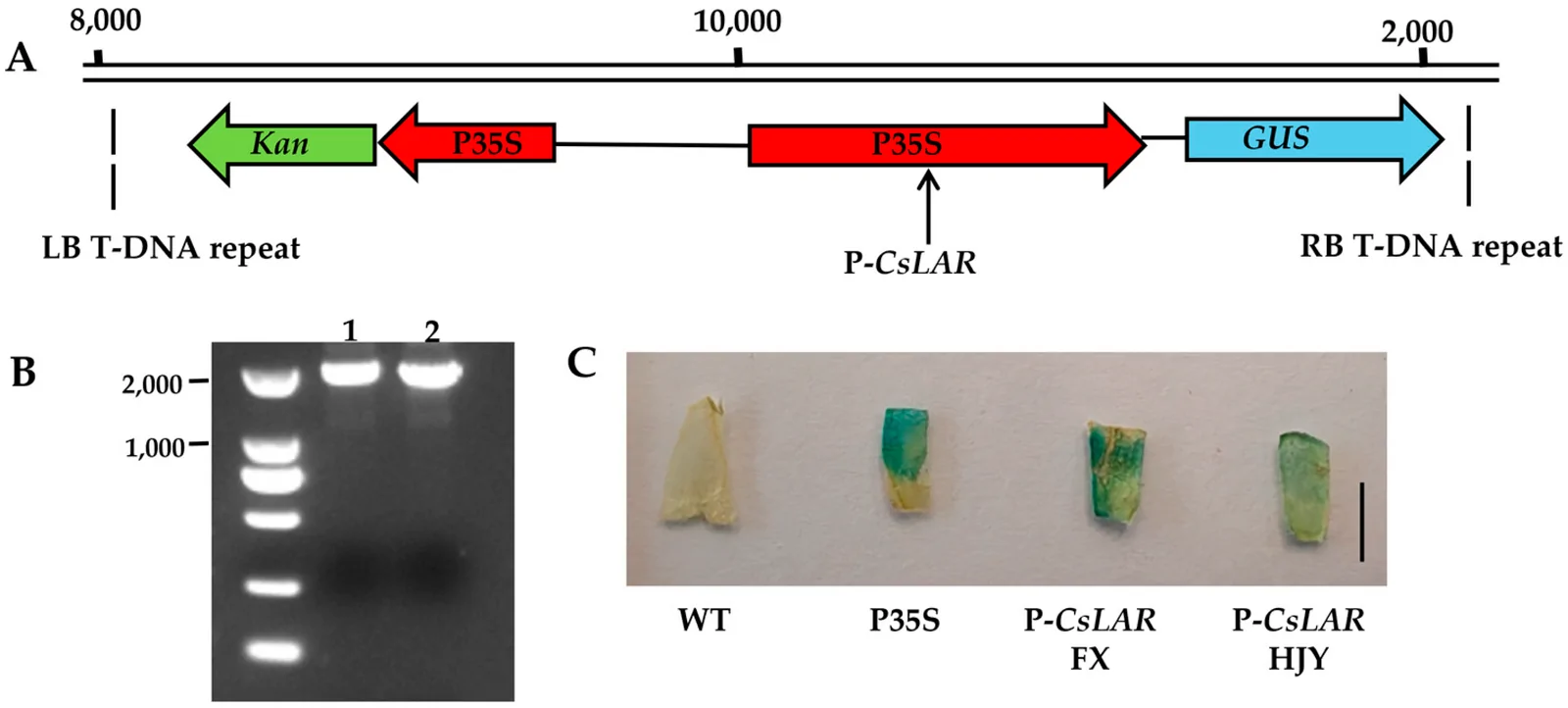
Analysis of *CsLAR* promoter activity. (**A**) Schematic diagram of the *P-CsLAR*::*GUS* plasmid structure. (**B**) PCR validation of recombinant plasmids carrying the *CsLAR* promoter from two tea cultivars. Lane 1: FX; Lane 2: HJY. (**C**) GUS staining of tobacco leaves transiently transformed with *P-CsLAR*::*GUS*. Scale bar = 1 cm.

Promoters play a key role in regulating gene expression. To investigate the regulatory mechanism of *CsLAR* expression and its association with catechin accumulation in two tea cultivars, an approximately 1900 bp fragment of the *CsLAR* promoter was isolated and subjected to activity analysis. PCR validation confirmed the successful construction of the P-*CsLAR*::*GUS* recombinant vector using the pCAMBIA3301 backbone (Figure 6B). Transient transformation assays in tobacco leaves demonstrated that the promoters from both cultivars exhibited transcriptional activity (Figure 6C). However, whether the observed differences in promoter activity between the two cultivars are attributable to variations in cis-elements or trans-acting factors remains to be elucidated.

#### 3.6.2. Comparative Analysis of *CsLAR* Promoter Elements Between FX and HJY

**Figure 7:**
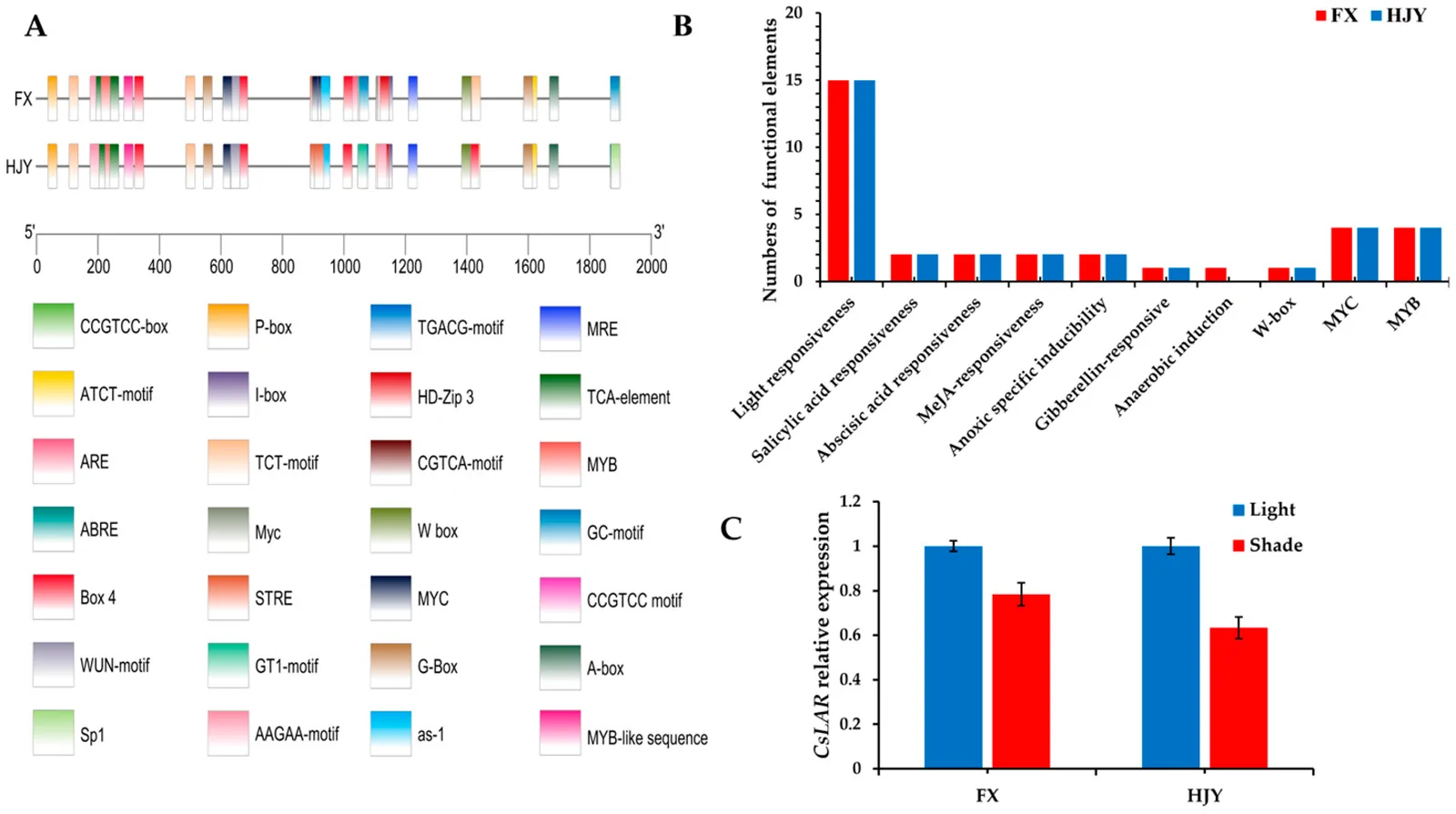
Analysis of the *CsLAR* promoter sequence. (**A**) Promoter element of the *CsLAR* gene in FX and HJY. (**B**)Numbers of cis-elements in the *CsLAR* promoters of FX and HJY. (**C**) Expression levels of *CsLAR* under normal light and shading conditions in both cultivars.

Variations in promoter architecture can influence gene expression by modulating cis-element composition. In this study, we performed a comparative analysis of the *CsLAR* promoter sequences from two tea cultivars, FX and HJY. The results revealed that both promoters contained the same number of functional elements, including those responsive to light and hormones, as well as binding sites for MYB and MYC transcription factors. Notably, an anaerobic response element (ARE) was detected exclusively in the FX promoter (Figure 7A,B).

Considering that HJY is a light-sensitive cultivar and both promoters share identical light-responsive elements, we applied shading treatments to both cultivars to examine potential differences in *CsLAR* gene expression patterns under a uniform cis-element background. Notably, following shading, *CsLAR* transcript levels decreased by 37% in HJY, whereas FX exhibited a more moderate reduction of 22% (Figure 7C). These results indicate that *CsLAR* expression in HJY is more sensitive to shading, suggesting that trans-acting factors may contribute to the differential regulation observed between the two cultivars.

### 3.7. Promoter Analysis of CsLAR Reveals Potential Regulatory Transcription Factors

**Figure 8:**
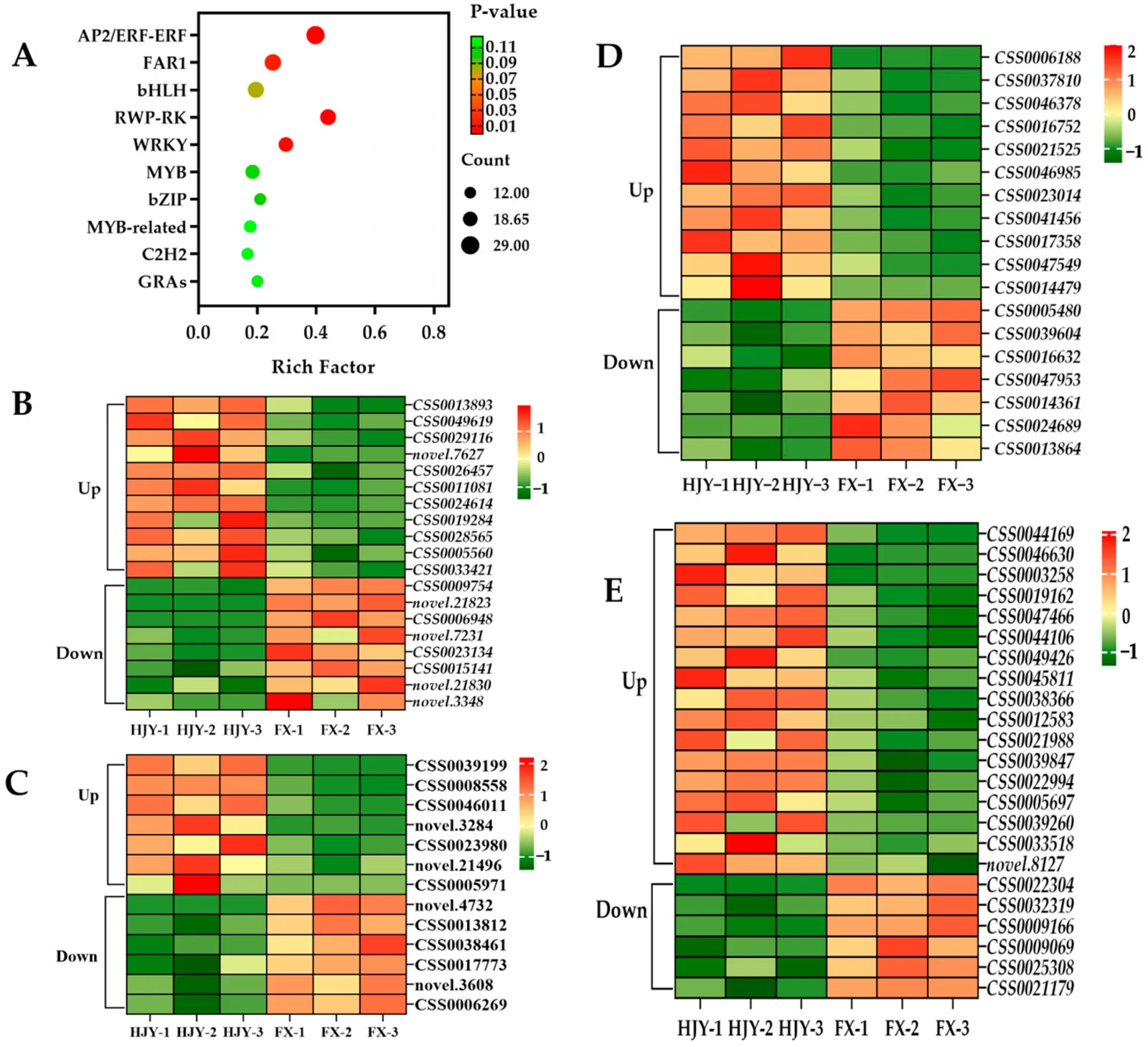
Analysis of differentially expressed transcription factors between FX and HJY. (**A**) Enrichment analysis of differentially expressed transcription factor families. (**B**) Heatmap of differentially expressed WRKY family transcription factors. (**C**) Heatmap of differentially expressed MYB-related family transcription factors. (**D**) Heatmap of differentially expressed MYB family transcription factors. (**E**) Heatmap of differentially expressed bHLH family transcription factors. Red and green indicate high and low expression levels, respectively.

Further analysis of the transcriptomic data between the two varieties identified 385 differentially expressed transcription factors (TFs), comprising 204 up-regulated and 181 down-regulated genes. Classification by family revealed that these differentially expressed TFs were predominantly enriched in the AP2/ERF, FAR1, bHLH, RWP-RK, WRKY, and MYB families (Figure 8A). Specifically, the MYB family included 19 differentially expressed members (11 up-regulated and 7 down-regulated) (Figure 8D), while the WRKY family comprised 19 differentially expressed genes (11 up-regulated and 8 down-regulated) (Figure 8B). Cis-element prediction revealed that the promoter region of the *CsLAR* gene (leucoanthocyanidin reductase) contains multiple specific binding cis-regulatory elements corresponding to these transcription factor families. This finding supports the hypothesis that the differentially expressed TFs may regulate *CsLAR* expression by binding to specific cis-elements in its promoter, thereby influencing catechin biosynthesis in HJY.

## 4. Discussion

Tea, a perennial woody species belonging to the Theaceae family, is characterized by a rich array of secondary metabolites that contribute to its complex flavor profile and determine the distinct sensory traits of different tea cultivars [26]. Among these, albino tea varieties are particularly favored by consumers due to their pronounced umami taste and reduced bitterness and astringency. HJY is a representative albino tea cultivar. To date, research on albino tea germplasms has primarily focused on the mechanisms underlying leaf etiolation or albinism and the associated developmental processes [19,27]. In recent years, the application of multi-omics technologies has focused on the composition and regulation of secondary metabolites, particularly the biosynthesis and accumulation of theanine, a non-proteinogenic amino acid closely associated with the umami and sweet tastes of tea [28]. However, beyond its umami character, low bitterness and astringency are also defining sensory attributes of HJY. Systematic investigations into the biochemical and molecular basis of these traits remain limited. In this study, we employed the green-leaf cultivar FX as a control to elucidate the mechanisms underlying the low bitterness and astringency traits of HJY.

### 4.1. Differential Metabolite Accumulation Is Primarily Associated with Flavonoids Rather Than Amino Acids

Albino tea is widely appreciated by consumers due to its pronounced fresh and brisk taste, coupled with low levels of astringency and bitterness. Studies have demonstrated that yellow-leaf tea cultivars exhibit higher amino acid accumulation. For instance, the amino acid content in buds of yellow cultivar ‘Zhonghuang 3’ was 3–65% higher than that of green cultivar ‘Zhongcha 108’ at different developmental stages [20]. Amino acids are core metabolites responsible for the fresh taste of tea. In this study, the total free amino acid content of HJY reached 32.79 mg/g, which was significantly higher than that of FX. This finding is consistent with previous reports and explains the superior fresh taste of HJY.

High amino acid content is primarily responsible for the fresh and brisk flavor profile of tea and may also partially mitigate astringency and bitterness [26]. However, flavonoids are the key determinants of bitterness and astringency in tea [29]. In this study, total flavonoid content was determined using the NaNO_2_-Al(NO_3_)_3_-NaOH method, and the results revealed that the flavonoid content in HJY was significantly higher than that in FX. Relevant studies have demonstrated that this colorimetric system mainly targets flavonols and their glycosides, while exhibiting weak response to catechins [30,31]. Moreover, previous investigations have revealed that flavonol levels are markedly elevated in yellow leaf tea cultivars [32,33]. Consistently, our widely targeted metabolome data also identified more up-regulated than down-regulated differential metabolites belonging to flavonols in HJY (Figure 3A). This may be the reason why the total flavonoid value determined by this colorimetric method is on the high side. More importantly, metabolomic analysis further uncovered that differential metabolites between the two cultivars were predominantly enriched in flavonoid-related pathways rather than amino acid metabolism. These findings suggest that the reduced astringency and bitterness perceived in HJY may not be merely due to masking by high amino acid content; rather, specific metabolic reprogramming of the flavonoid pathway and the altered accumulation pattern among different flavonoid subclasses may collectively contribute to its characteristic flavor.

### 4.2. The Unique Accumulation Pattern of Catechins Is Critical to Flavor Formation in HJY

Catechins, a major subclass of flavan-3-ols, are primary determinants of the bitterness and astringency of tea and serve as key indicators of tea quality [34]. Previous studies have demonstrated that tea catechins (EC, ECG, EGC, EGCG, etc.) can be recognized by human bitter taste receptors of the TAS2R family and activate bitter-sensing pathways [35]. For astringency, EGCG reduces oral-tissue lubrication, and the reduction in lubrication is positively correlated with astringency intensity [36]. Although non-esterified catechins also induce astringent sensation, they hardly alter oral lubrication and exhibit higher astringency thresholds [8,37]. In the present study, compared with the control cultivar FX, HJY displayed significantly decreased accumulation of non-esterified catechins, whereas esterified catechins remained unchanged. Given that non-esterified catechins have been proven to be the key factors responsible for bitterness and astringency, we hypothesize that the reduction in the accumulation of these compounds in HJY might be a metabolic characteristic, related to the low bitterness and astringency of this product. Moreover, glycosylated catechin derivatives exhibit substantially reduced bitterness and astringency compared to their aglycone forms [38,39]. Notably, a significant increase in the accumulation of glycosylated catechin derivatives was observed in HJY, which may further contribute to the reduction of undesirable taste characteristics. Collectively, these metabolic alterations are consistent with the formation of a flavor profile characterized by low bitterness and astringency and enhanced briskness in HJY. However, to directly confirm this relationship, precise sensory evaluation is still required. Importantly, esterified catechins also serve as important bioactive constituents in tea [40]. The maintained levels of these compounds in HJY ensure the retention of its health-promoting properties, thereby conferring a dual advantage of improved sensory quality and preserved functional value.

### 4.3. Molecular Regulation of the Distinctive Catechin Accumulation Pattern in HJY

Analysis of transcriptome sequencing data revealed that the expression level of *CsLAR* was significantly downregulated in HJY compared to FX [41]. Previous studies have confirmed that overexpression of the *CsLAR* gene can significantly increase catechin content in plants, which is consistent with the findings of this study [42,43]. These observations suggest that the differential accumulation of catechins in HJY may be regulated, at least in part, by the transcriptional activity of the *CsLAR* gene.

Gene transcription is regulated by promoter regions, and transcription factors can modulate transcription by binding to cis-acting elements within promoters [44,45]. This study analyzed the promoter elements of the *CsLAR* gene from two varieties and investigated their regulation by transcription factors. By cloning and comparing the promoter sequences of the *CsLAR* gene, the results revealed only one key difference in the promoter elements between the two varieties. Both promoters contained light-responsive elements but responded differently to shading treatment, with HJY being more sensitive. After shading, the expression of the *CsLAR* gene decreased by 37% in HJY, compared to a 22% decrease in FX. Therefore, it is hypothesized that the expression of this gene is also regulated by transcription factors.

Among the more than 60 transcription factor families identified in higher plants, WD40, MYB, WRKY, bHLH, and bZIP are the most common and crucial families. Transcriptome analysis in this study revealed significant differential expression of transcription factors from these families between HJY and FX. Notably, all of these transcription factor families have corresponding binding sites within the *CsLAR* promoter region. Previous studies have reported that WRKY70 can bind to the *CsLAR* promoter and regulate its expression in tea plants [46]. During the ripening process of strawberries (*Fragaria* × *ananassa* Duch. and *Fragaria chiloensis* (L.) Mill.), MYB1 affects the accumulation of anthocyanins by participating in the trans-activation of the *LAR* promoter [47]. In Chinese plum (*Prunus salicina* Lindl.), the MYB family transcription factor PsaMYB700 activates *LAR* expression, and its transcript level is significantly positively correlated with (+)-catechin accumulation in fruits [48]. In this study, the expression of transcription factors *WRKY70* (CSS0023134) and *MYB1* (CSS0016632) was also significantly downregulated in HJY, further supporting their potential involvement in the regulation of *CsLAR*-mediated catechin accumulation. Nevertheless, to identify the key transcription factors directly responsible for modulating *CsLAR* expression in HJY, additional molecular validation, such as yeast one-hybrid assays and dual-luciferase reporter experiments, is required. Such investigations will provide a basis for the precise improvement of tea quality through targeted genetic manipulation.

## 5. Conclusions

This study systematically elucidated the differential mechanisms underlying catechin accumulation and flavor formation in the tea cultivars HJY and FX through an integrated metabolomic and transcriptomic approach. Metabolomic analysis revealed that the levels of bitter-tasting non-esterified catechins (C, EC, EGC) were significantly lower in HJY than in FX, whereas no significant differences were observed for esterified catechins such as EGCG and GCG. These findings provide a metabolic basis that may be associated with the reduced bitterness and astringency characteristic of HJY. Transcriptomic data further demonstrated that *CsLAR*, a key gene involved in catechin biosynthesis, was significantly downregulated in HJY, correlating with the decreased accumulation of non-esterified catechins and suggesting a pivotal role for *CsLAR* in regulating catechin accumulation.

Regarding the regulatory mechanism, although the promoter structure and distribution of light-responsive cis-elements of *CsLAR* were highly conserved between the two cultivars, shading treatment resulted in a more pronounced downregulation of *CsLAR* expression in HJY, suggesting heightened light sensitivity in this cultivar. A total of 385 differentially expressed transcription factors—including members of the MYB and WRKY families—were identified, and their corresponding binding motifs were predicted within the *CsLAR* promoter region. These results imply that the differential expression of *CsLAR* between cultivars is likely mediated by transcription factor binding to the promoter, thereby modulating flux through the catechin biosynthesis pathway and influencing flavor development. From a multi-omics perspective, this study identifies metabolic and transcriptional features potentially associated with low bitterness and astringency in HJY, offering a theoretical foundation and technical support for the breeding of tea cultivars with improved flavor profiles.

## Figures and Tables

**Table 1:** Primers used for promoter amplification and real-time q-PCR analysis.

Name	Primer Sequence 5′→3′
P-*CsLAR*	F: AACTGCAGTCTGTATCATCAGCTAATTGTC
	R: CATGCCATGGTTACGTACCTCCCTCTAGTCCT
*GAPDH*	F: TTGGCATCGTTGAGGGTCT
	R: CAGTGGGAACACGGAAAGC
q*CsLAR*	F: TCTTCAAGACAAAGGCGCTAA
	R: CCAAACTCCGATGGCAAAA

**Table 2:** Statistics of differentially accumulated metabolites in FX versus HJY.

	Total Metabolites	Different Metabolites	Up Regulated	Down Regulated
Number	1228	601	383	218

**Table 3:** Counting of DEGs between FX and HJY.

	Total DEGs	Up Regulated DEGs	Down Regulated DEGs
Number	9099	4368	4731

## Data Availability

The original contributions presented in this study are included in the article. Further inquiries can be directed to the corresponding authors.

## Abbreviations

The following abbreviations are used in this manuscript:

C	Catechin
EC	Epicatechin
EGC	Epigallocatechin
EGCG	Epigallocatechin gallate
CHS	Chalcone synthase
CHI	Chalcone isomerase
F3H	Flavonoid 3-hydroxylase
F3′5′H	Flavonoid 3′,5′-hydroxylase
DFR	Dihydroflavonol 4-reductase
ANS	Anthocyanidin synthase
LAR	Leucoanthocyanidin reductase
UGGT	Galloyl-1-O-β-D-glucosyltransferase
ECGT	1-O-galloyl-β-D-glucose O-galloyltransferase
